# Visual imagery skills and risk attitude

**DOI:** 10.1038/s41598-022-25627-y

**Published:** 2022-12-10

**Authors:** Cathy Zeng, David Fielding, Ronald Peeters, Dennis Wesselbaum

**Affiliations:** 1 Department of Economics, University of Otago, PO Box 56, Dunedin, 9054 New Zealand; 2 Global Development Institute, University of Manchester, Oxford Rd, Manchester, M13 9PL UK

**Keywords:** Human behaviour, Decision

## Abstract

Several of Kahneman and Tversky’s seminal works in the 1970s found evidence of the importance of framing in decision making under risk. They hypothesized that imaginability (visual imagery ability) may play an important role in the evaluation of subjective probabilities. However, the impact of visual imagery ability on choice under risk has not yet been explored. This is the main purpose of our study. In an online experiment, we collected participants’ visual imagery ability using the Vividness of Visual Imagery Questionnaire and their risk attitude using two choice-based risk elicitation tasks. Participants made their risk decisions either in an environment where risk was visualized (visual frame) or not (non-visual frame), and were randomly assigned to one of the two decision frames. Our results suggest that neither visual imagery ability nor decision frame has a substantial impact on risk attitude.

## Introduction

In decision making under risk, visual imagery skills may influence how individuals evaluate probabilities. The seminal paper by Tversky and Kahneman in 1973^1^, fundamental in the development of their prospect theory^2,3^, discusses how imaginability can affect subjective probability: “*Imaginability plays an important role in the evaluation of probabilities in real-life situations. The risk involved in an adventurous expedition, for example, is evaluated by imagining contingencies with which the expedition is not equipped to cope. If many such difficulties are vividly portrayed, the expedition can be made to appear exceedingly dangerous, although the ease with which disasters are imagined need not reflect their actual likelihood. Conversely, the risk involved in an undertaking may be grossly underestimated if some possible dangers are either difficult to conceive of, or simply do not come to mind*”. This text suggests two sources of variation in the strength with which images come to mind: first the framing of the task (“if many such difficulties are vividly portrayed”) and second the visual imaginative capacity of the subject (“if some possible dangers are either difficult to conceive of, or simply do not come to mind”).

Visual imagery is defined by the American Psychological Association (APA) as “mental imagery that involves the sense of having “pictures” in the mind”. Within the fields of behavioral and cognitive psychology, the quality of visual imagination has been investigated in relation to sensory systems, cognitive processes (such as short-term memory^4^), and perceptual decision making (such as for threatening cues^5^). Given that imaginability was suggested as a key component of subjective probability by Kahneman and Tversky 50 years ago, it is surprising that the role of visual imaginative capacity in the context of choice under risk (and choice behavior in general) has not been explored in earlier research.

In this study, we explore the possible impact of visual imaginative capacity on risk-taking behavior. To this end, we conducted an online experiment in which we collected data on imaginative capacity and risk behavior of 239 participants. In order to measure imaginative capacity, we used the Vividness of Visual Imagery Questionnaire (VVIQ) developed by Marks in 1973^6^. To measure risk, we used the first principal component^7^ of decisions reported in the risk elicitation tasks developed by Holt and Laury and by Eckel and Grossman^8,9^. Using these data we test for a potential impact of visual imagery ability on choice behavior. Since the existing literature has reported gender differences in both visual imagery skills and risk behavior, we control for gender differences.

As noted above, Tversky and Kahneman suggested the potential importance of framing as well as of imaginative capacity. Although there may be important implications of how risk is presented (for instance, for public health messaging during a pandemic), the literature relating the presentation of risk to risky choice is rather meager^10,11^. In order to examine the role of framing, we randomly assigned participants to one of two different decision frames: in one of these, the lotteries between which the participants were to choose are presented non-visually; in the other, they were presented visually.

## Experimental design

Our experiment consisted of five parts. In Parts 1 and 2, participants’ risk attitudes were elicited using the Holt and Laury and the Eckel and Grossman procedures respectively^8,9^. In Part 3, participants were presented with a version of the Allais Paradox^12^, to check whether their behavior violated expected utility theory. Part 4 measured the quality of participants’ imagination using the Vividness of Visual Imagery Questionnaire (VVIQ) developed by Marks^6^, and Part 5 consisted of five short demographic questions. Details about these five parts are publicly available on the Open Science Framework (OSF; doi: 10.17605/osf.io/9s7rv) and accessible via https://osf.io/9s7rv/.

**Figure 1:**
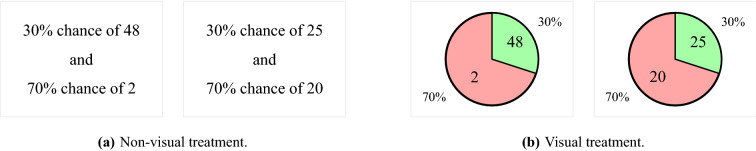
The third choice problem in the Holt and Laury task.

We implemented a $2\times 2$ between-subjects factorial design, resulting in four experimental treatments. The treatments varied along two dimensions. The first treatment variation was in the presentation of the lottery choices in Parts 1–3: the presentation was either non-visual or visual. In the non-visual treatment, lottery choices were presented in text form, as displayed in Fig. 1a, while in the visual treatment they were presented in graphical form, as displayed in Fig. 1b. Participants encountered either the non-visual or the visual treatment in all three parts. The second treatment variation was the way in which we presented the VVIQ, with participants being asked to fulfil the imagining exercise with their eyes either open or closed. The questionnaire itself and all other instructions were identical across the two VVIQ treatments.

There is no consensus in the literature about the shape of the distribution of choices in the risk elicitation tasks, and so we do not use parametric tests of the association between our variables of interest. Instead, we rely on the Wilcoxon–Mann–Whitney test. The precise power of this test depends not only on the sample size, but also on the shape of the distribution of the variables and on the proportion of subjects in each group. Nevertheless, if the effect size in the population is 0.5 standard deviations, if the variables are normally or uniformly distributed, and if the groups have approximately equal sizes, then (with a two-sided test) a total sample size of about 150 is required to ensure an 80% probability of rejecting the null of no effect using a 5% critical value^13^. (With an exponential distribution, the required sample size is slightly smaller.) Given the possibility of some sample attrition (for reasons described below), it is prudent to work with a sample size of about 200 in order to ensure a power of 80%, and this is the norm in the experimental economics literature^14–16^. After having presented our main results, we will also report an ex-post power analysis to calculate how large the sample would need to be to have 80% power, were the effect sizes in the population as large as those estimated in our actual sample.

Data were collected in a single session on Thursday, December 16, 2021. We recruited 240 participants via *Prolific*, an online participant recruitment platform (we had three selection criteria: age 18–75, fluent in English, and using a desktop, laptop, or tablet). From here, they were redirected to *Qualtrics*. Informed consent was obtained before they were presented the tasks. Participants received £1.25 for completing the study and had the opportunity to earn a bonus of up to £5 depending on their decisions in the first three tasks and chance. Each participant was paid according to the outcome of their chosen lottery in a randomly selected choice situation within a randomly selected risk task. They received £0.05 for each unit of payoff relating to the outcome of the lottery. All randomizations were computerized and applied at the individual level. All experiments were performed in accordance with relevant guidelines and regulations. Data collection was anonymous, participation was voluntary, and no deception was used. This study was approved by the University of Otago Human Ethics Committee (reference code D21/415) and pre-registered with the AEA RCT registry (ID number AEARCTR-0008709).

As online experiments become increasingly common in economics and other social sciences, there have been questions about the validity of data obtained by this means. However, several studies have shown that experiments held in face-to-face laboratories replicate well when held online^17–19^. We chose to recruit participants using *Prolific* since this platform “combines good recruitment standards with reasonable cost, and explicitly informs participants that they are recruited for participation in research”^20^. Additionally, it has been shown that, using several measures, *Prolific*’s data quality is higher than that of other frequently used participant recruitment platforms^21,22^. For our study, *Qualtrics* recorded our response quality as 100%. The median completion time was 9 min and 19 s (average 11 min and 56 s), around 95% of participants spent at least 5 min completing the experiment, and around 68% more than 8 min.

## Data and variables

Our dataset comprising 239 observations (one observation was excluded from the dataset as the participant did not complete the experiment) has a good gender balance, with 102 participants identifying as male at birth (43%) and 137 as female at birth (57%). 126 of the 239 participants are in the first age bracket (18–24), 70 in the second (25–34), 28 in the third (35–44), 9 in the fourth (45–54), 4 in the fifth (55–64), and 1 in the sixth (65+); one participant preferred not to reveal their age. The average earnings were £2.63 (including a fixed participation fee of £1.25). Given the small number of participants in the last three age brackets, we restrict our dataset to the 224 participants in the first three age brackets for our analyses. These remaining participants were approximately uniformly distributed over the four treatment variations (see Table 1 for a breakdown).

**Table 1:** Observations by treatment.

	Non-visual	Visual	Total
Eyes open	53	58	111
Eyes closed	58	55	113
Total	111	113	224

The most important variables in our study are risk attitude and visual imagery skills, measured by the VVIQ. The creation of these variables is described in the following sections. Table 2 presents the descriptive statistics.

**Table 2:** Descriptive statistics.

	HL	EG	VVIQ
Non-visual and eyes open	0.48 (0.21)	0.41 (0.33)	0.68 (0.12)
Non-visual and eyes closed	0.46 (0.21)	0.47 (0.34)	0.70 (0.14)
Visual and eyes open	0.55 (0.22)	0.35 (0.33)	0.66 (0.20)
Visual and eyes closed	0.53 (0.24)	0.33 (0.34)	0.70 (0.13)
All treatments	0.51 (0.22)	0.39 (0.34)	0.69 (0.15)

Mean values are reported with standard deviations in parentheses. The labels heading the columns refer to the variables elicited in Tasks 1–4, which are explained in the remainder of this section.

### Risk attitude

In the Holt–Laury task, participants make ten choices, each between two lotteries: one lottery is a safe option and the other is a risky option. We did not ask participants to choose a single point at which they would switch from the safe lottery to the risky lottery; rather, we constructed the variable *HL* as the fraction of cases in which participants made the risky choice: $\textit{HL}=0$ indicates no risky choices and $\textit{HL}=1$ indicates all risky choices.

In the Eckel–Grossman task, participants make one choice out of five lotteries, ranging from a riskless lottery to a very risky lottery. Individuals who are extremely risk averse would choose the sure bet, Lottery 1, while a moderately risk averse individual would choose one of the intermediate bets, Lotteries 2–4; a risk-neutral or risk-seeking individual would choose Lottery 5. In creating the variable *EG*, we assigned each lottery a value from 0 to 1, with 0 being the safe choice and 1 the riskiest choice, so that there was a linear relationship between the value of the variable and the riskiness of the lottery chosen in the task.

**Figure 2:**
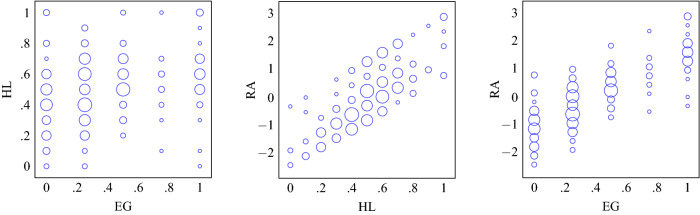
Relation between the risk variables.

**Figure 3:**
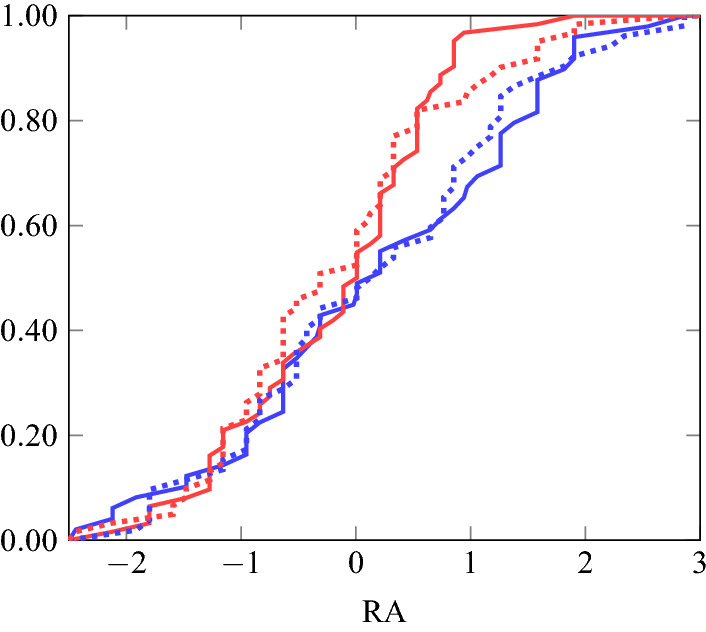
Cumulative distributions of *RA* for the non-visual (solid) and visual (dotted) treatments for males (blue) and females (red).

The scatter plot on the left of Fig. 2 shows the relationship between the two constructed risk variables. They have a significant positive correlation (Pearson: $\rho =0.2598$; $p=.0001$). Since the two variables are likely to be noisy, we conducted a principal component analysis, measuring attitude towards risk (*RA*) as the first principal component of *HL* and *EG*^7,23^. (The non-standardized Cronbach’s alpha for the variables *HL* and *EG* is 0.3846. The two variables are derived from 11 items in total: 10 for *HL* and 1 for *EG*. The Spearman-Brown prediction for the alpha of 0.3846 with 11 items equals 0.775, which is an acceptable value.) As with the underlying risk measures, higher values of *RA* should be interpreted as representing more willingness to take risks. The two scatter plots on the right of Fig. 2 show the relationship between the output (*RA*) and the two inputs (*HL* and *EG*). There is a high correlation of the output variable with each of the input variables (Pearson: $\rho =0.7937$; $p=0.0000$) and *RA* explains 63% of the variation in *HL* and *EG*. Figure 3 presents cumulative distributions of our risk measure, *RA*, disaggregated by frame and gender.

### Visual imagery skills

The VVIQ asks participants to rate how clearly they are able to picture various situations in their mind’s eye^6^. Most research using the VVIQ as a measure of visual imagery has participants take the questionnaire twice, once with their eyes open and once with their eyes closed. For the practical reason of keeping the online experiment short and thus maintaining high attention levels, we decided to have participants only take the questionnaire once. In some existing studies, tests with eyes-open data lead to rejection of a null but tests with eyes-closed data do not^24^, while in other studies the reverse is true^25^. This indicates that some characteristics are more strongly related to eyes-open scores and others more strongly related to eyes-closed scores. In the absence of a literature to indicate which is more relevant to risk attitudes, we decided to use both versions and randomly assign participants to either treatment.

For both the eyes-open and the eyes-closed versions, following common practice, we constructed the visual imagery skills variable, *VVIQ*, by adding up participants’ ratings in each task, giving a total score ranging from 16 (low vividness) to 80 (high vividness). We then normalized this to the unit interval, applying a linear transformation so that 0 indicates the lowest possible level of visual imagery skills and 1 the highest^26^. That is, a score *X* on the scale between 16 and 80 is recorded as $\textit{VVIQ}=(X-16)/64$ in our results. Many studies have documented a high internal consistency reliability of the VVIQ score^27^ and several studies have provided neuroscientific evidence in its favor^4,28,29^.

**Figure 4:**
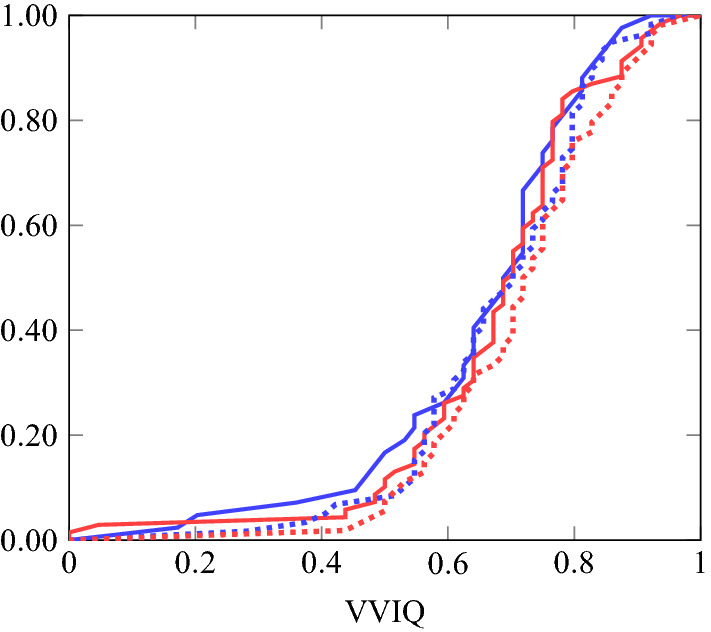
Cumulative distributions of *VVIQ* elicited with eyes open (solid) and eyes closed (dotted) for males (blue) and females (red).

Figure 4 presents our VVIQ measure disaggregated by eyes open/closed and gender. A Wilcoxon–Mann–Whitney test indicates no significant gender differences in the VVIQ score. This contrasts with the literature, which finds significant differences in VVIQ scores between males and females^30^. Yet, consistent with findings in the literature, females on average show higher visual imagery skills than males (the mean value of *VVIQ* is 0.6751 for males and 0.6964 for females).

To allow for noisiness in the data, we create a categorical variable following Marks^6^, allocating each participant to one of three groups: “low visualizers”, “medium visualizers”, and “high visualizers”. The categorization is applied separately across sexes (males and females) and VVIQ treatments (eyes open and eyes closed)^31^. Within each of the four groups, for which the number of observations is reported in Table 3, the bottom third is classified low visualizers and the top third is classified as high visualizers; in the case of ties, participants are allocated to the more extreme category. All low visualizers have a VVIQ value below 0.6797 (that is, a score of at most 59 on the range from 16 to 80) and all high visualizers a value above 0.7265 (that is, a score of at least 63 on the range from 16 to 80); further details are provided in the online appendix (accessible via OSF). Note, however, that our labels “low” and “high” are not to be interpreted qualitatively as “poor” and “good”^32^.

**Table 3:** VVIQ categories.

	VVIQ treatment		VVIQ skill category			
Gender	Eyes open	Eyes closed	Low visualizers	Medium visualizers	High visualizers	Total
Male	42	59	34	33	34	101
Female	69	54	42	35	46	123
Total	111	113	76	68	80	224

We then pool the eyes-open and eyes-closed groups, so that we have a single sample for each gender (male and female) and each frame (non-visual and visual). This method allows for potential differences in scale interpretation across the eyes-open and eyes-closed treatments. Table 3 presents the number of observations for each of the six groups. In the analysis related to the impact of visual imagery skills on risk attitude, the medium groups are discarded, and the low and high skill categories are compared. The difference in the *VVIQ* values between the upper quartile of the low visualizer group ($\textit{VVIQ}=0.6094$) and the lower quartile of the high visualizer group ($\textit{VVIQ}=0.7813$) is slightly more than one standard deviation based on the full sample ($\text {std}=0.1517$).

## Results

**Figure 5:**
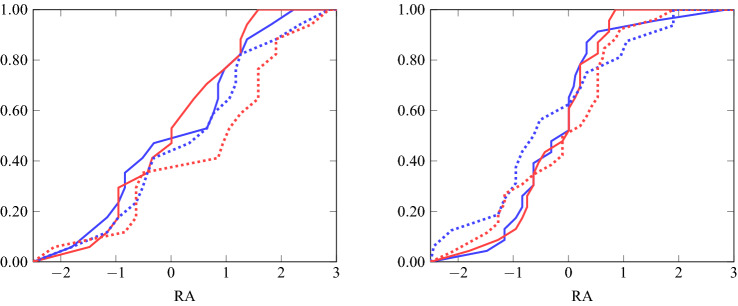
Cumulative distributions of *RA* in non-visual (blue) and visual (red) frames for groups with high (solid) and low (dotted) visual imagery skills for males (left) and females (right).

For each of the two decision frames, we have four subgroups of individuals based on gender and visual imagery skills. The sample sizes for the different groups are given in Table 4. Figure 5 shows eight cumulative distributions over risk attitude (i.e., the variable *RA*) in these groups. The left graph shows these for the non-visual (blue) and visual (red) treatments for groups with high (solid) and low (dotted) visual imagery skills for males. The right graph shows this for females. The results reported below are based on comparisons between the distributions in these graphs.

**Table 4:** Group sizes.

	Male		Female	
	Low visualizers	High visualizers	Low visualizers	High visualizers
Non-visual frame	17	17	26	23
Visual frame	17	17	16	23

We note in passing that we cannot reject normality of the variable *RA*. Nevertheless, we report only results from a non-parametric Wilcoxon–Mann–Whitney test. This is for two reasons: first, our initial research proposal envisaged only a non-parametric test; second, principal components analysis has a tendency to produce a normal distribution, even when (as is the case with *HL* and *EG*) the underlying variables are not normally distributed. The properties of parametric tests using principal components have not been extensively researched. We also conduct an ex-post power analysis using bootstrapping techniques. That is, for each of the eight groups we select at random twelve members, resulting in 48 observations for each of the two compared groups, and apply a two-sided Wilcoxon–Mann–Whitney test. By balancing the sample selection we prevent the outcome being driven by other potentially confounding factors. We replicate this sampling and testing procedure a million times and record the resulting *p* values. We report the median and mean of these *p* values, and the percentage of cases where the *p* value is below one of three critical threshold levels (1%, 5% and 10%). In other words, we estimate what the power of our test statistic would be, if the effect size in the population were equal to the one we have estimated. The bootstrap also facilitates an estimation of the sample size required for 80% power with an effect of this size. Finally, for exploratory purposes, we conduct Wilcoxon–Mann–Whitney tests for different subgroups. Detailed results of these tests are available in our online appendix (accessible via OSF). However, we acknowledge that results for subgroups may be underpowered.

First, we investigate the relationship between visual imagery skills and risk attitude. This concerns the comparison between the high and low visual imagery ability categories; that is, the comparison between the solid and dotted distributions in the figure.

### Result 1

 There is no evidence for an impact of visual imagery skills on risk attitude.

*Evidence.* Based on a two-sided Wilcoxon–Mann–Whitney test using data pooled across gender and decision frames, we cannot reject equality of risk attitude between low and high visualizers: $p=0.2499$.

The balanced bootstrapping procedure produces a median (mean) *p* value of 0.3275 (0.3872). The test produces a *p* value below 0.10 in only 23% of cases, a value below 0.05 in only 14% of cases, and a value below 0.01 in only 4% of cases.

*Further exploratory findings* We also applied the Wilcoxon–Mann–Whitney tests to different subgroups: only participants in the non-visual frame, only participants in the visual frame, only males, only females, only males in a single frame, and only females in a single frame. For none of the eight subgroups is there a significant difference, even when we do not adjust the test statistic for multiple hypotheses testing. While low visualizers took more risk on average than high visualizers (this is true of every subgroup except females in the visual frame), the difference is not statistically significant.

Our next result concerns how visualization of the decision frame affects risk attitude; that is, the comparison between the blue and red distributions in the figure.

### Result 2

 There is no evidence for an impact of visual framing on risk attitude.

*Evidence.* Based on a two-sided Wilcoxon–Mann–Whitney test using data pooled across gender and visualizer groups, we cannot reject equality of risk attitude between the non-visual and the visual decision frame: $p=0.7276$.

The balanced bootstrapping procedure results in a median (mean) *p* value of 0.4791 (0.4864). The test produces a *p* value below 0.10 in only 11% of cases, a value below 0.05 in only 6% of cases, and a value below 0.01 in only 1% of cases.

*Further exploratory findings.* Like for Result 1, we also applied the Wilcoxon–Mann–Whitney tests to the different subgroups. As in Result 1, there was no significant difference for any subgroup. Across the whole sample (and for five of the eight subgroups), participants in the non-visual frame took more risk on average than those in the visual frame, but this difference is statistically insignificant.

Finally, we compare risk-taking behavior across genders.

### Result 3

 There are gender differences in risk attitude: males take more risk than females.

*Evidence.* Based on a two-sided Wilcoxon–Mann–Whitney test using data pooled across decision frames and visual imagery skills, we can reject equality of risk attitude between genders: $p=0.0046$. This is consistent with the existing literature^33,34^.

The balanced bootstrapping procedure results in a median (mean) *p* value of 0.0238 (0.1023). The test produces a *p* value below 0.10 in 74% of cases, a value below 0.05 in 62% of cases, and a value below 0.01 in 37% of cases.

*Further exploratory findings.* We also applied the Wilcoxon–Mann–Whitney tests to the different subgroups. In each subgroup, males take more risk on average than do females. For the three subgroups involving low visualizers in the non-visual decision frame, we find the gender difference to be statistically significant at 5% level, but only if we do not adjust the test statistic for multiple hypotheses testing. There is no significant difference for the other five subgroups.

Our sample size was intended to ensure 80% power with an effect size in the population equal to 0.5 standard deviations. In fact, the pooled standard deviation in our sample was 1.1206, and the observed effect sizes for the three results were 0.1881, 0.0350 and 0.4735 standard deviations respectively. Bootstrapping indicates that if the effects in the population were this large, rejection of the null with 80% power using a 5% confidence interval would require sample sizes of 1410, 40,863 and 225, respectively. With our current sample sizes, means and standard deviations, we find a power of 21%, 4% and 81%, respectively.

## Discussion and conclusion

Several of Kahneman and Tversky’s seminal works in the 1970s suggested that framing and imaginability (visual imagery ability) may play an important role in decision making under risk. Based on the data collected in an online experiment, we find no evidence of any significant impact of visual imagery ability or of visual framing on risk choices. However, we do find significant gender differences.

The fact that we find no evidence of an association between the capacity for visual imagery and choices about risk does not mean that all individuals follow the same decision-making processes. The same disclaimer applies to the comparison of risky choices across decision frames: the way risk is framed may still affect the decision-making process. As shown in the literature, both the capacity for visual imagery and environmental context can affect perceptual decision making, i.e. the way sensory information guides choices^5^. However, our experiment was not designed to isolate such effects, as we measured participants’ choices rather than their underlying preferences. Likewise, in the absence of underlying preference data, we are not able to make normative statements about which individuals make correct or incorrect decisions in different decision frames.

In this paper, the focus is on risk choices, as captured by the variable *RA*, rather than the two individual risk elicitation methods, but the data we collected does allow for such an analysis and comparison. If the outcome variable is the Holt–Laury task (that is, *HL* instead of *RA*), then all three of our main results remain the same. (See the online appendix for details.) For the Eckel–Grossman task (*EG*), Result 1 remains the same, but Results 2 and 3 do not. Using *EG*, there is a significant association of risk attitudes with the form in which the task was presented (visual or non-visual), but no significant association with gender. Identifying the reasons for the differences between the *HL* and *EG* results requires further research.

The main limitation of our study is that the sample is not large enough for comparisons across subgroups, or for investigation of the risk behavior of individuals with aphantasia (those with no mind’s eye) or with hyperphantasia (whose mental images are as vivid as actual visual perception). While aphantasia has been associated with impaired imagery across multiple sensory domains (including auditory, olfactory and gustatory imagination)^35,36^, and with intellectual disability^37^, we are not aware of a study that relates it to choice behavior. Such an investigation would require a more targeted participant recruitment^38^. Finally, a larger sample would facilitate difference-in-difference analyses between subgroups and to identify possible non-linear effects.

Our research was conducted in the gains domain only. In many practical applications, such as insurance decisions, loss aversion may have more impact, given the potential for choices to be driven by fear of loss. Results showing that visual imagery skills amplify emotional response to reading a fearful scenario^39^ suggest that the capacity for visual imagery may influence how one views loss in decision making under risk when choices are presented visually. Lang’s bio-informational theory of emotional imagery has led to a literature examining the relationship between visual imagery and emotion^40^. While the literature was initially focused on fear, it has since expanded to other emotions, such as anxiety^41,42^. We consider a replication of our study in the loss domain with a focus on loss attitude to be worthwhile as a follow-up study.

## Data Availability

Survey details, online appendix, data files, and *Stata* and *Matlab* codes used for the analyses are publicly available on the Open Science Framework (OSF; doi: 10.17605/osf.io/9s7rv) and accessible via https://osf.io/9s7rv/.
